# BurnoutEnsemble: Augmented Intelligence to Detect Indications for Burnout in Clinical Psychology

**DOI:** 10.3389/fdata.2022.863100

**Published:** 2022-04-05

**Authors:** Ghofrane Merhbene, Sukanya Nath, Alexandre R. Puttick, Mascha Kurpicz-Briki

**Affiliations:** ^1^Applied Machine Intelligence Research Group, Department of Engineering and Information Technology, Bern University of Applied Sciences, Bern, Switzerland; ^2^Institute for Research in Open, Distance and eLearning (IFeL), Swiss Distance University of Applied Sciences, Brig, Switzerland

**Keywords:** burnout, natural language processing, machine learning, augmented intelligence, ensemble classifier, psychology

## Abstract

Burnout, a state of emotional, physical, and mental exhaustion caused by excessive and prolonged stress, is a growing concern. It is known to occur when an individual feels overwhelmed, emotionally exhausted, and unable to meet the constant demands imposed upon them. Detecting burnout is not an easy task, in large part because symptoms can overlap with those of other illnesses or syndromes. The use of natural language processing (NLP) methods has the potential to mitigate the limitations of typical burnout detection *via* inventories. In this article, the performance of NLP methods on anonymized free text data samples collected from the online forum/social media platform Reddit was analyzed. A dataset consisting of 13,568 samples describing first-hand experiences, of which 352 are related to burnout and 979 to depression, was compiled. This work demonstrates the effectiveness of NLP and machine learning methods in detecting indicators for burnout. Finally, it improves upon standard baseline classifiers by building and training an ensemble classifier using two methods (subreddit and random batching). The best ensemble models attain a balanced accuracy of 0.93, test F1 score of 0.43, and test recall of 0.93. Both the subreddit and random batching ensembles outperform the single classifier baselines in the experimental setup.

## 1. Introduction

Stress at the workplace is an increasingly relevant topic. In a study involving almost 10,000 working adults in eight territories throughout Europe, it was found that 18% of the respondents feel stressed daily, and three out of ten participants feel so stressed that they have considered finding a new job (ADP, 2018). A Swiss study (SECO, 2015) estimates that 24.2% of employees feel often or always stressed at their workplace, while 35.2% feel mostly (22.2%) or always (13%) exhausted at the end of the working day. In the latter group, 25.5% still feel exhausted the next morning, a circumstance which, if prolonged, can lead to various health hazards. Studies from the United States give the same indication. The Stress in America's Report of 2019 by the American Psychological Association shows that Americans consider a healthy stress level at an average of 3.8 (scale ranging from 1 to 10, where 10 is “a great deal of stress” and 1 is “little or no stress;”) however, they report having experienced an average stress level of 4.9 (American Psychological Association, 2019).

This stress can lead to workplace burnout. In 2019, the WHO included burnout in the 11th Revision of the International Classification of Diseases (ICD-11) as a syndrome.^1^ In particular, during the pandemic crisis, burnout in the healthcare sector was an important issue: it has been shown, for instance, that the COVID-19 crisis has had an overwhelming psychological impact on intensive care workers (Azoulay et al., 2020).

Identifying burnout syndrome is complex because symptoms can overlap with other diseases or syndromes (Jaggi, 2019). In particular, the overlap between depression and burnout is an important subject of scientific discussion, e.g., (Schonfeld and Bianchi, 2016). In clinical intervention and field research, burnout is typically detected *via* the use of *inventories*. Potential burnout patients fill out a psychological test, usually in the form of a questionnaire with scaled-response answers (e.g., not at all, sometimes, often, very often). Although such inventories are used in most studies and are well-established in the clinical environment, major limitations have been identified. For example, in personality inventories, participants are liable to fake their results, e.g., (Holden, 2007). They may adapt their responses in high-stake situations in order to increase their chances for the desired outcome (Lambert, 2013). A further issue with inventories is known as *extreme response bias* (ERB); some respondents will tend to choose (or avoid) only the highest or the lowest options in such tests (Greenleaf, 1992; Brulé and Veenhoven, 2017). It has also been shown that on self-reported tests for subjective well-being, the respondent's mood during testing sometimes contributes as a predictor (Diener et al., 1991). Furthermore, defensiveness (the denial of symptoms) and social bias can influence the outcome of inventories (Williams et al., 2019).

A potential way to mitigate the existing and well-known problems with inventories is to explore the use of free text questions or transcribed interviews. Previous studies have demonstrated promise in such methods (Burisch, 2014), but, in practice, the manual effort of analyzing the resulting data often results in untenable overhead costs. Fortunately, recent developments in the field of natural language processing (NLP) make approaches using such unstructured textual data feasible. It has been shown that computational linguistic markers can be used to predict depressivity of the writer (Havigerová et al., 2019).

Existing work applying NLP to psychology focuses on the identification of indicators for different types of mental health disorders by using data obtained from social media, comprising the majority of available research in this area. For example, such work concentrates on suicide risk assessment (Morales et al., 2019), (Just et al., 2017), depression (Moreno et al., 2011), (Schwartz et al., 2014), post-partum depression (De Choudhury et al., 2013), (De Choudhury et al., 2014), or different mental health signals (Coppersmith et al., 2014). In some cases, data from Reddit online forums have been used, for example, to detect mental health disorders (Thorstad and Wolff, 2019), anxiety (Shen and Rudzicz, 2017), or depression (Tadesse et al., 2019).

However, very little work exists in the field of burnout detection. Burnout detection in data extracted from issues and comments posted within software development tools have been studied (Mäntylä et al., 2016). The authors used the valence-arousal-dominance (VAD) model to study burnout risk in a corporate setting. This model distinguishes three emotions: *valence* (“the pleasantness of a stimulus,”) *arousal* (“the intensity of emotion provoked by a stimulus,”) and *dominance* (“the degree of control exerted by a stimulus”) (Warriner et al., 2013). To measure burnout risk, the metric is based on low valence and dominance and high arousal (Mäntylä et al., 2016). In other work, a first attempt to detect burnout based on patient and expert interviews in the German language were done; it was found that a combination of NLP and machine learning techniques in this field leads to promising results (Nath and Kurpicz-Briki, 2021).

In the context of earlier work focused on gathering data from social media websites and the study of mental health conditions, this work extends state-of-the-art predictive models in the field while focusing specifically on detecting indicators for burnout in data collected from Reddit. It aims to develop the base technology for potential new directions in tool development for clinical psychology. Herein, the authors emphasize that this work is oriented toward the approach of augmented intelligence rather than artificial intelligence (Rui, 2017); instead of replacing clinical professionals, it strives toward technology that empowers humans in the decision-making process, providing input to be considered in human decision-making.

The work in this article addresses the following objectives:

- It evaluates whether NLP methods applied to free text are an effective means to detect indicators for burnout, compared to a control group using general text samples, and a control group with depression-related texts.
- In particular, it investigates how the use of an ensemble classifier can leverage the accuracy of such methods.
- Furthermore, the approach is compared to single machine learning classifiers such as logistic regression.

This article is structured as follows: first, the materials and methods used in this work are discussed. In particular, this includes data collection, the characteristics of the datasets used in the experiments, and the experimental setup. Then, the results are presented, first for single classifier models and then for the ensemble models. Finally, the results are discussed and an outlook on potential future work is provided.

## 2. Materials and Methods

### 2.1. Reddit Data Collection

On Reddit, users can organize posts based on a subject, so-called *subreddits*, which are online micro communities dedicated to a particular topic. Reddit has the advantage of allowing the possibility to create micro communities *via* subreddits. As a result, in addition to topics such as gaming and music, there are thriving communities dedicated to various mental health topics, such as depression, anxiety, and bipolar disorder. In particular, there is a subreddit dedicated to burnout; unfortunately, the number of entries was too low at the time of our data collection to provide a sufficiently large dataset. However, users discuss the subject of burnout in various other subreddit threads. One can thus collect textual data related to burnout by scraping Reddit for burnout-related posts. In this work, praw (Boe, 2011), a Python Reddit API Wrapper, was used to extract submissions with the keyword “burnout” and its different variations, such as “burnout,” “burn out,” “burned out,” “burning out,” “burnt out,” “burn-out,” etc., 1,536 such submissions were found.

However, the word *burnout* also widely occurs in other contexts, such as “The tires are burnt out.” It is also frequently used in informal discussions, such as having *game burnout* or *music burnout*. It was therefore necessary to isolate submissions describing burnout in the professional or educational context. A total of 677 submissions satisfying these conditions were manually identified. The replies to the selected submissions were also collected, as they were likely to contain posts by other users describing their experiences with burnout. This increased the size of the dataset to 23,371 posts. However, not all of the posts and replies were relevant to professional or educational experiences with burnout. Therefore, 352 instances were extracted manually that describe burnout experiences from a first-person perspective. This formed the test group for the data classified as *burnout*.

To create the first control group, the *no burnout* dataset, our method employed the strategy described in Shen and Rudzicz (2017). Namely, 17,025 posts from a variety of subreddits were collected: “askscience”, “relationships”, “writingprompts”, “teaching”, “writing”, “parenting”, “atheism”, “christianity”, “showerthoughts”, “jokes”, “lifeprotips”, “writing”, “personalfinance”, “talesfromretail”, “theoryofreddit”, “talesfromtechsupport”, “randomkindness”, “talesfromcallcenters”, “books”, “fitness”, “askdocs”, “frugal”, “legaladvice”, “youshouldknow”, and “nostupidquestions”, Since a number of these collected posts consisted of empty or very little text, all posts consisting of fewer than 100 characters were dropped, resulting in a final *no burnout* dataset consisting of 13,216 posts.

The second control group, the *depression* dataset, was collected from the subreddit for depression and contains 979 posts. As for burnout, only entries using the first-person perspective were selected.

The authors emphasize that no information concerning user identity (e.g., username or age) was collected.

### 2.2. Datasets for Experiments

Using the raw data consisting of 13,216 posts labeled *no burnout* (control group), 352 labeled *burnout*, and 979 labeled *depression*, four datasets for use in the experiments were compiled. Dataset statistics are presented in Table 1.

**Dataset 1: Burnout vs. No Burnout (BNB):** It combines the 13,216 *no burnout* posts with the 352 *burnout* posts, resulting in a highly unbalanced dataset of size 13,568.
**Dataset 2: Burnout vs. No Burnout (Balanced) (BNB-Balanced):** Balanced dataset of 704 posts, of which, 352 posts are selected from the *no burnout* dataset through random sampling (without replacement). Additionally, an equal number of 352 posts are added from the *burnout* data.
**Dataset 3: Burnout vs. No Burnout (No Keywords) (BNB-No-Keywords):** It is obtained from Dataset 2 by removing the keywords from the *burnout* dataset that were used during data collection to search for burnout-related posts: “burnout,” “burn-out,” “burning out,” etc.
**Dataset 4: Burnout vs. Depression (BD):** Balanced dataset of 704 entries, of which 352 posts are selected from the *depression* dataset through random sampling (without replacement). Further, an equal number of 352 posts are added from the *burnout* data.

**Table 1:** Dataset statistics.

**Dataset Name**	**No. of Samples**	**Mean Text Length (chars)**	**Std. Dev of Text Length**	**Test Group %age**	**Control Group %age**
1. Burnout vs. No Burnout(BNB)	13,568	1158	1451	2.6%	97.4%
2. Burnout vs. No Burnout
(Bal.) (BNB-balanced)	704	867	850	50%	50%
3. Burnout vs. No Burnout
(No KWs)(BNB-no-keywords)	704	863	846	50%	50%
4. Burnout vs. Depression (BD)	704	1009	905	50%	50%

*“Control” refers to either no burnout or depression, while test refers to burnout*.

### 2.3. Vectorization

The spacy^2^ Python NLP-library was used in order to vectorize text data for use in our NLP models. Each Reddit post was tokenized using the pre-trained en_core_web_sm English language pipeline and converted into a 500-dimensional bag-of-words vector, which simply counts the occurrences of each of the 500 most commonly appearing words in the text corpus.

### 2.4. Experimental Setup

#### 2.4.1. Single Classifier Models

The following experiment was repeated on Datasets 1–4. The feature set consisted of the vectorized Reddit posts, each labeled with either 1 (burnout) or 0 (no burnout/depression). Using a 70-30% training-test split^3^ and 10-fold cross-validation (CV), a variety of classifier models was trained: logistic regression, Support Vector Machine (SVM) (with linear, RBF, degree 3 polynomial and sigmoid kernels), and random forest. Each model's performance was measured by using the mean CV accuracy and F1 scores averaged across all folds, as well as the (balanced) accuracy, F1, and recall scores on the test data. It was chosen to specifically include recall as a metric because, in a real-world setting, it would be important to capture all possible *burnout* samples (recall = 1), even at the expense of a larger number of false positives (see Section 4.5 for further discussion).

#### 2.4.2. Ensemble Classifier Models

Ensemble classifiers allow aggregating the decisions of several single classifier models. The ensemble methods presented in this work closely resemble a method known as UnderBagging (Barandela et al., 2003). Each ensemble is built according to the template below.

*Ensemble model template:*

- The ensemble consists of *n* submodels.^4^
- Each submodel is trained with 10-folds CV on a balanced dataset of 492 posts.
- These datasets share the same 246 *burnout* samples but contain pairwise disjoint sets of *no burnout* samples.
- The prediction of the whole ensemble is determined by voting, i.e., for a given test sample, a label of *burnout* is predicted if the voting >*p*% of the submodels classify the sample as *burnout*.^5^ Otherwise, the sample is classified as *no burnout*.

**Figure 1:**
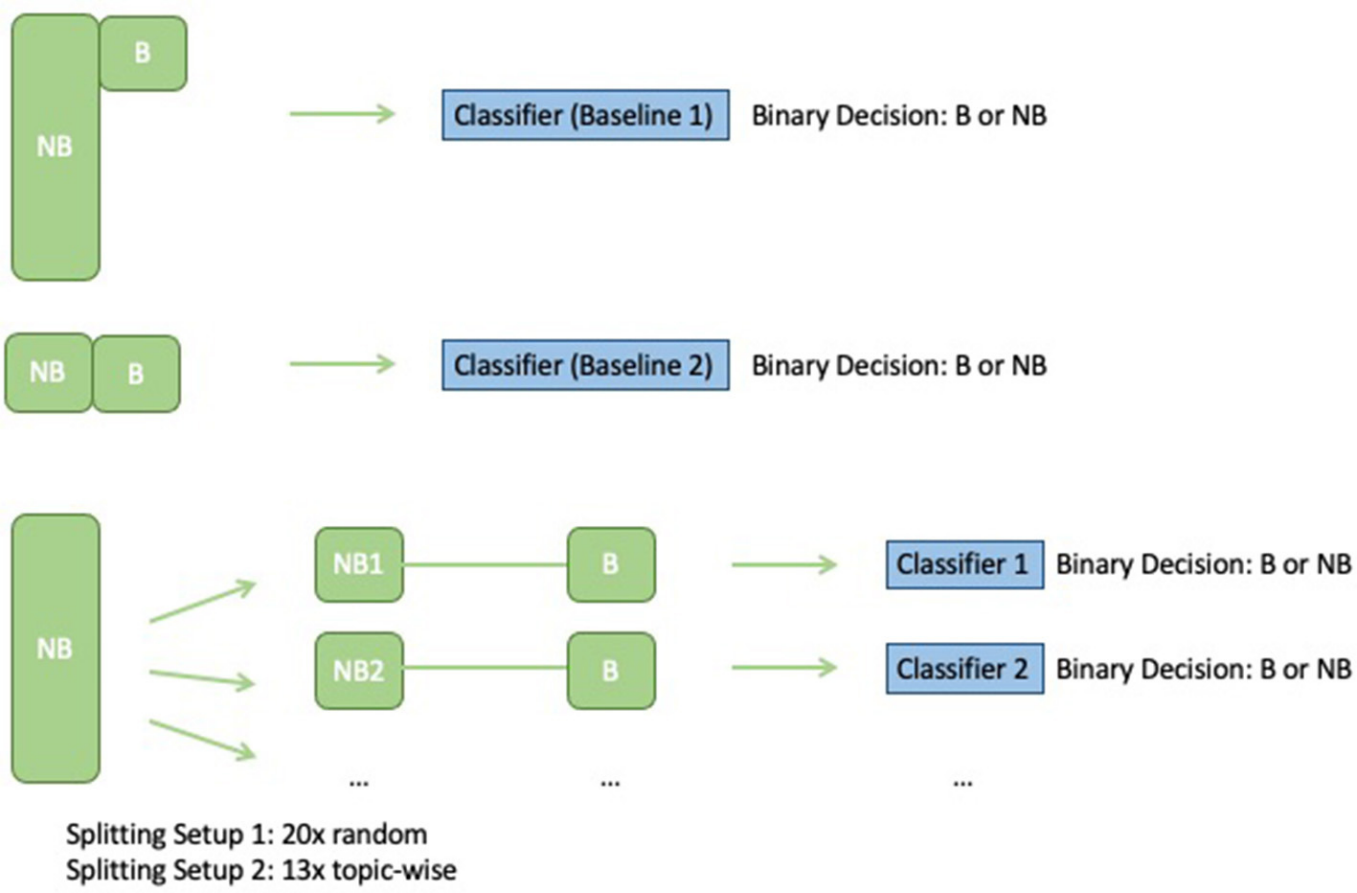
Training the baselines vs. training ensembles on balanced batches.

The classifier type of the submodels was restricted to logistic regression, which demonstrated the most consistent performance in our initial experiments, although RBF, linear SVMs, and random forests also showed promise. Figure 1 shows a depiction of our ensemble setup, along with a comparison to the two baseline models to which the ensemble results were compared:

- **Baseline 1:** Logistic regression classifier trained *via* 70–30% train-test split on the unbalanced Dataset 1 (BNB).
- **Baseline 2:** Logistic regression classifier trained on a balanced dataset obtained by randomly sampling 246 *no burnout* samples and combining them with the 246 *burnout* samples used for training.

**Figure 2:**
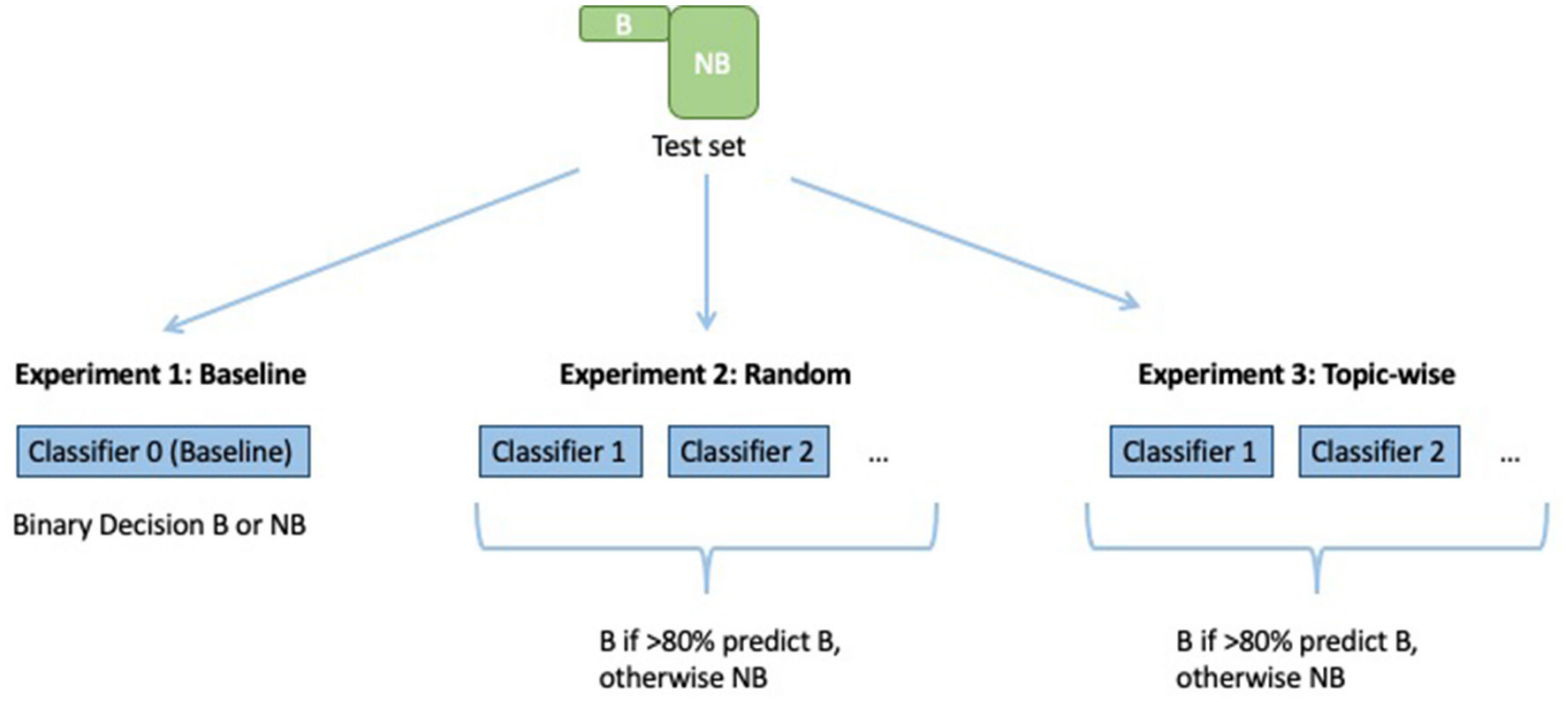
Computing predictions on the test set, with a voting threshold of *p* = 0.8 for example purposes.

Figure 1 depicts the setup for training the baseline classifier (logistic regression on the full unbalanced training set) and the ensemble classifiers, and Figure 2 depicts how each model makes predictions on the test data.

**Figure 3:**
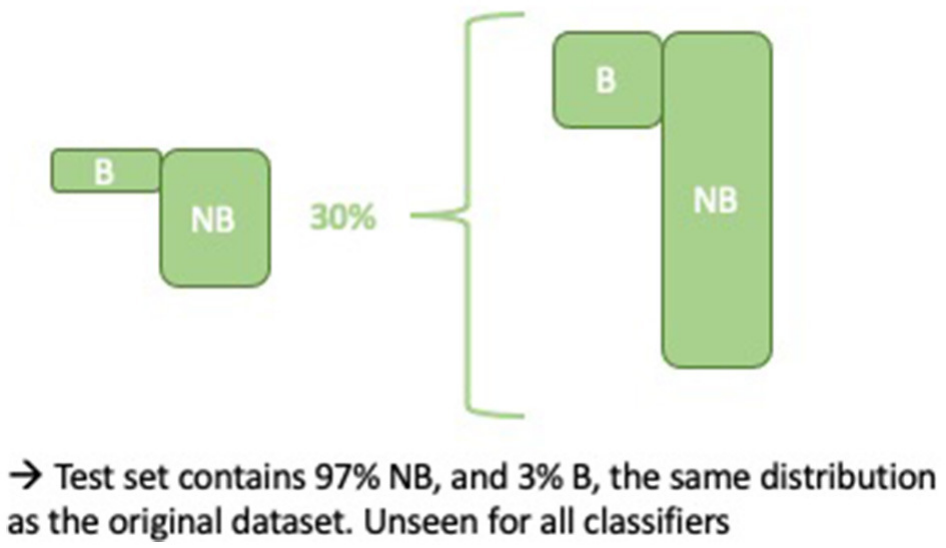
Constructing the unbalanced test set of 4,071 samples.

Training and test data were allocated according to a 70-30% split. This was done in a stratified manner, i.e., the *no burnout* and *burnout* class distribution in the training and test data were approximately equal to the distribution in Dataset 1 (BNB) (as shown in Figure 3). Note that the same test data were used for both baselines and ensembles.

Two types of data batching were tested in our ensembles (Ensemble 1 and Ensemble 2, see description below) and the following metrics were measured:

- *Mean CV accuracy:*^6^ Computed by first taking the mean CV accuracies for each submodel over the 10 folds, followed by averaging over the *n* submodels.
- *Mean CV F1 (macro):* Identical with F1 in place of accuracy.
- *Mean test balanced accuracy*: The balanced accuracy on test data averaged across the *n* submodels.
- *Mean test F1 (macro)*: Identical for F1.
- *Mean test recall*: Identical for recall.
- The corresponding SDs of the above three test metrics.

**Ensemble 1: Random sample batching:**

The *random sample batching* ensemble was trained using *n* = 20 batches, each consisting of 246 randomly sampled (without replacement) posts from the *no burnout* training samples concatenated with 246 burnout training samples to create BNB-balanced datasets.

**Ensemble 2: Batching by subreddit:**

The *subreddit batching* ensemble was trained by creating a balanced dataset corresponding to each of the subreddits appearing in the *no burnout* training data for which at least 246 samples had been collected. There were *n* = 17 such subreddits in total.

The effect of changing the voting threshold *p* on the ensemble performance was also tested. Values of *p* = 0.4, 0.5, 0.6, 0.7, 0.8, and 0.9 were evaluated.

## 3. Results

### 3.1. Single Classifier Models

#### 3.1.1. Burnout vs. No Burnout

The results of the single classifier experiments on Dataset 1 (Burnout vs. No Burnout BNB) are displayed in Table 2. The *Baseline* row corresponds to a model that predicts the label *no burnout* for all samples. Such a model achieves 97% accuracy due to the class imbalance in Dataset 1 (BNB). Indeed, accuracy is a misleading measure in such a situation: all classifiers in this experiment demonstrated an accuracy of approximately 97% despite large differences in performance. For this reason, balanced accuracy provides a more meaningful metric for model performance.

**Table 2:** Results for Burnout vs. No Burnout – Dataset 1 (BNB) (no. test samples = 4071).

**Model**	**Mean CV Bal. Acc**.	**Mean CV F1**	**Test Bal. Acc**.	**Test F1**	**Test Recall**
Logistic Regression	0.72	0.48	0.75	0.49	0.50
SVM Linear	0.72	0.40	0.75	0.45	0.51
SVM RBF	0.51	0.04	0.51	0.03	0.01
SVM Poly Degree 3	0.55	0.16	0.56	0.18	0.12
SVM Sigmoid	0.57	0.23	0.56	0.21	0.12
Random Forest	0.50	0.02	0.51	0.04	0.02
Baseline	0.50	0.0	0.50	0.0	0.0

*Baseline refers to a model predicting no burnout for all samples. The mean CV statistics are computed by taking an average of overall 10 folds cross-validation (CV) during training*.

Only logistic regression and SVM linear demonstrated significant improvement over the baseline, although roughly 50% of burnout samples were incorrectly classified as *no burnout*.

#### 3.1.2. Burnout vs. No Burnout (Balanced)

Here, the results of classifiers trained using Dataset 2 (BNB-balanced) are presented. Aside from the SVM poly degree 3 classifier, the models in Table 3 appear to demonstrate good performance.^7^ It was noted that these results are dependent on the random sample of *no burnout* data points that are used to construct Dataset 2 (BNB-balanced). While random forest classifiers demonstrated the best performance in this instance, there were also cases in which logistic regression performed best. For the best models, accuracies and F1 scores approximately distributed between 0.90 and 0.97 were observed.

**Table 3:** Results for Burnout vs. No Burnout (Balanced)—Dataset 2 (BNB-balanced) (no. test samples = 234).

**Model**	**Mean CV Accuracy**	**Mean CV F1**	**Test Accuracy**	**Test F1**
Logistic regression	0.91	0.91	0.87	0.88
SVM Linear	0.89	0.89	0.84	0.85
SVM RBF	0.88	0.88	0.89	0.89
SVM Poly degree 3	0.60	0.35	0.60	0.41
SVM Sigmoid	0.85	0.85	0.82	0.82
Random Forest	0.92	0.92	0.88	0.89

*The mean CV statistics are computed by taking an average of overall 10 folds CV during training*.

#### 3.1.3. Burnout vs. No Burnout (No Keywords) (BNB-No-Keywords)

The data collection process applied in this work explicitly searches for burnout-related keywords. It is, therefore, possible that trained models identify the presence of such keywords as a key defining feature for posts belonging to the *burnout* class. The effect of the presence of such keywords was measured, and it was tested whether they provided a significant basis for the models' predictions. Therefore, all keywords related to burnout were removed from Dataset 2 (BNB-balanced) to obtain Dataset 3 (BNB-no-keywords) and the experiment was repeated. The corresponding results are displayed in Table 4. As one might expect, the removal of keywords resulted in decreased model performance. However, the decrease was not very important, providing evidence that the presence of keywords is not an overly important factor in any of our other experiments.

**Table 4:** Results for Burnout vs. No Burnout (no keywords)—Dataset 3 (BNB-no-key-words) (no. test samples = 234).

**Model**	**Mean CV Accuracy**	**Mean CV F1**	**Test Accuracy**	**Test F1**
Logistic regression	0.88	0.88	0.86	0.87
SVM Linear	0.85	0.85	0.82	0.83
SVM RBF	0.85	0.83	0.85	0.86
SVM Poly degree 3	0.59	0.34	0.59	0.40
SVM Sigmoid	0.79	0.80	0.81	0.82
Random Forest	0.88	0.88	0.87	0.88

*The mean CV statistics are computed by taking an average of overall 10 folds CV during training*.

#### 3.1.4. Burnout vs. Depression (BD)

In this experiment, as shown in Table 5, the Burnout vs. Depression dataset (Dataset 4, BD) was classified by using the models described previously. Again, it was found that logistic regression and SVM linear performed best, with random forest following closely. The datasets are balanced, and the random baseline for both accuracy and F1 score is set at 50%.

**Table 5:** Results for Burnout vs. Depression—Dataset 4 (BD).

**Model**	**Mean CV Accuracy**	**Mean CV F1**	**Test Accuracy**	**Test F1**
Logistic regression	0.87	0.87	0.84	0.82
SVM Linear	0.84	0.85	0.82	0.78
SVM RBF	0.84	0.85	0.78	0.77
SVM Poly degree 3	0.59	0.42	0.65	0.43
SVM Sigmoid	0.82	0.82	0.78	0.76
Random Forest	0.85	0.86	0.81	0.80

*The mean CV statistics are computed by taking an average of overall 10 folds CV during training*.

Although these models perform well, an across-the-board decrease of roughly 0.04 points is observed compared to the results listed in Table 3.

### 3.2. Ensemble Models

Tables 6, 7 record metrics and statistics that pertain exclusively to the submodels and not to the overall ensembles. They are meant as a means to compare the performance of the individual submodels to that of the ensembles (Table 8).

**Table 6:** Submodel CV averages.

**Model**	**Mean CV Accuracy**	**Mean CV F1**
Random Batching	0.91	0.90
Subreddit Batching	0.96	0.96

**Table 7:** Submodel test statistics (no. test samples = 4,071).

**Model**	**Mean Test Bal. Accuracy**	**Std. Dev. Test Bal. Acc**.	**Mean Test F1**	**Std. Dev. Test F1**	**Mean Test Recall**	**Std. Dev. Test Recall**
Random Batching	0.91	0.01	0.35	0.02	0.91	0.02
Subreddit Batching	0.78	0.08	0.13	0.04	0.96	0.02

**Table 8:** Ensemble vs. baseline performance (Threshold *p* = 80%, no. test samples = 4,071).

**Model**	**Test Bal. Acc**.	**Test F1**	**Test Recall**
Random Batching Ensemble	0.91	0.56	0.84
Subreddit Batching Ensemble	0.93	0.34	0.95
Baseline 1: Unbalanced LR	0.75	0.49	0.50
Baseline 2: Random Undersampling LR	0.90	0.33	0.91

Table 6 records the average CV performance metrics over the submodels within each of the ensemble classifiers. Recall that each of these submodels is a logistic regression classifier trained on a balanced dataset. The *Mean CV Accuracy* and *Mean CV F1* columns in Table 6 are thus comparable to the corresponding columns in Table 3.

Table 7 records the average test statistics for the submodels. The *Mean test Bal. Acc*. and *Mean test F1* columns refer to the average performance of the submodels on the unbalanced test set consisting of 4,071 samples, of which 106 belong to the *burnout* class. It also provides the corresponding SDs. The random batching submodels were much more consistent than the subreddit batching submodels, the latter of which demonstrated greater variance and lower average balanced accuracy and F1 score while achieving higher recall scores.

The test results reveal the limitations of the previously presented non-ensemble models trained on balanced data. Those models appeared to demonstrate very good performance on unseen test data (Table 3), but were tested on small balanced test sets consisting of only 234 posts. In comparison, the mean test F1 scores in Table 7 are relatively low, which shows that the high test performance observed in Table 3 does not imply similar performance on the unbalanced dataset of 4,071 samples. The high test recall in Table 7 indicates that the test F1 scores are primarily reduced due to low precision, i.e., a relatively large number of false positives.

The mean test metrics in Table 7 corresponding to random batching give an indication of how the logistic regression model trained on Dataset 2 (BNB-balanced) (Table 3) would perform on the large unbalanced test dataset used in our ensemble experiments. Note that the performance of the balanced data model depends on the random sample of 352 *no burnout* posts used to construct Dataset 2 (BNB-balanced), and significant fluctuations in performance were observed depending on the sample, encapsulated in the SDs recorded in Table 7. Indeed, the pursuit of ensemble approaches presented in this work was driven partially by the desire for a model with more stable performance. Effectively, Table 7 portrays the average performance of logistic regression models trained on balanced No Burnout vs. Burnout data over *n* = 20 disjoint random samples of *no burnout* data. It was observed that the submodels trained *via* subreddit batching demonstrated lower performance on the test data than those trained *via* random batching.

Table 8 shows the test results of the two ensemble models. The logistic regression (LR) model trained on Dataset 1 (BNB) was used as a first baseline, which demonstrated the best overall performance on the unbalanced test data among the single-model classifiers. As a second baseline, a single logistic regression classifier trained on a balanced dataset obtained by randomly undersampling from the *no burnout* class was considered, as was done to construct Dataset 2 (BNB-balanced). The model demonstrated performance similar to the averages recorded in Table 7.

The ensemble models demonstrated substantially improved balanced accuracy and recall relative to the baseline unbalanced LR model. However, the unbalanced LR model achieved the second-highest F1 score. Both ensembles and the baseline random undersampling LR demonstrated similar performance, with the random batching ensemble exhibiting a trade-off between F1 and recall. In comparing Tables 7, 8, one can see that the random batching ensemble demonstrates a relatively modest performance improvement over the submodels composing it. On the other hand, the subreddit batching ensemble performs markedly better than its component submodels.

**Figure 4:**
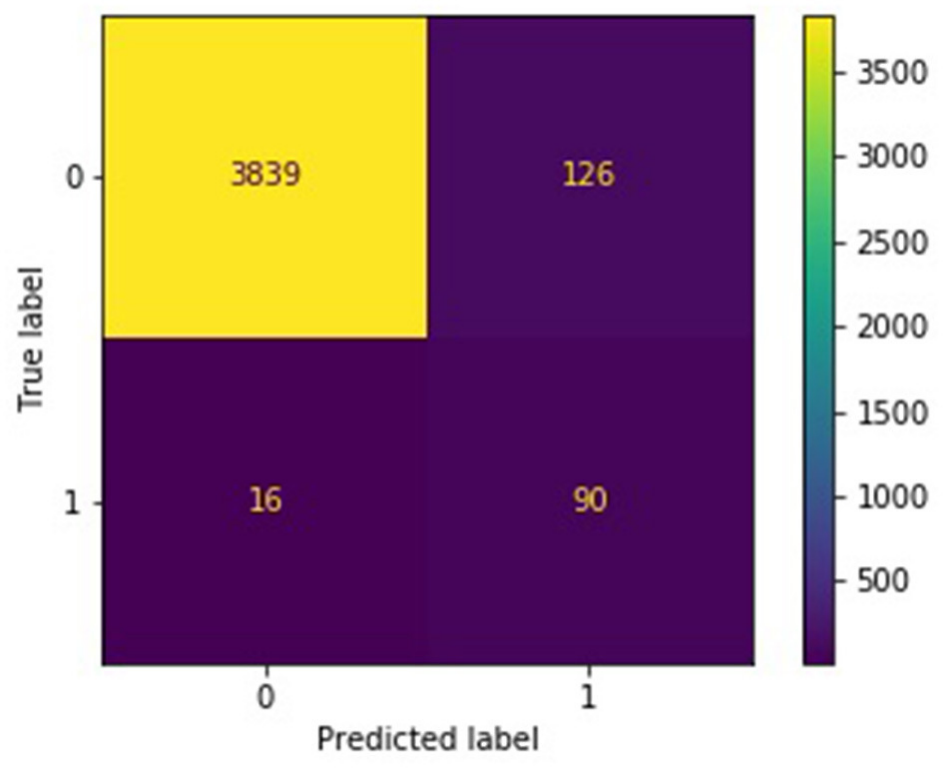
Confusion matrix for random batching ensemble, *p* = 0.8.

**Figure 5:**
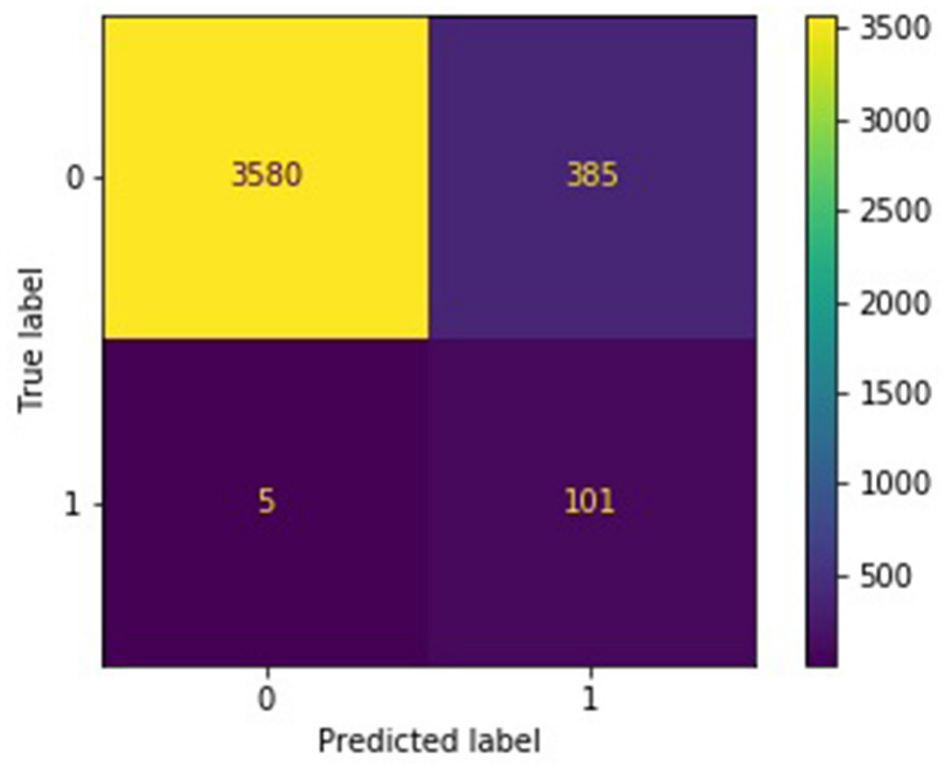
Confusion matrix for subreddit batching ensemble, *p* = 0.8.

The confusion matrices in Figures 4, 5 describe the distribution of the ensemble models' test predictions. Both the submodels and ensembles had test recall scores near 1. However, the high recall of the submodels came at the cost of a large number of false positives. It was observed that each submodel identified approximately 400–500 (random batching) to 1,000–3,000 (subreddit batching) test samples as belonging to the *burnout* class, whereas the correct number was 106. In contrast, with a majority vote threshold of *p* = 80%, the ensemble models placed 216 (random batching). There were 486 (subreddit batching) test samples in the burnout class while maintaining a recall score close to 1. The majority vote ensemble rule is thus an effective method for eliminating false positives while preserving true positives.

**Figure 6:**
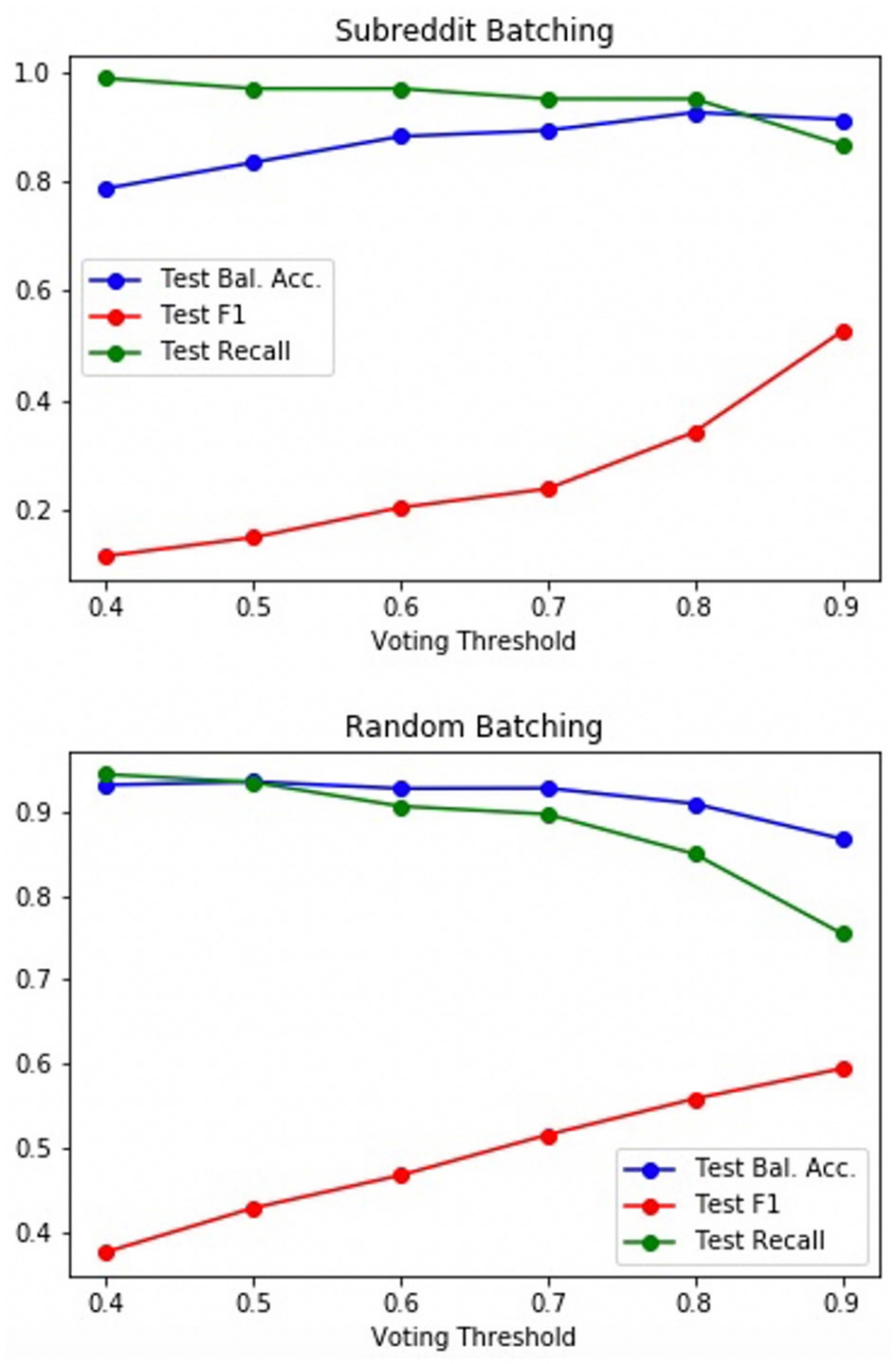
Ensemble performance vs. voting threshold.

The effect of modifying the voting threshold on performance was also tested. The results are depicted in Figure 6.

With random batching, a trade-off between recall/balanced accuracy and F1 score was experienced; while subreddit batching demonstrated a trade-off between recall and F1 score, balanced accuracy, and F1 score could be simultaneously improved, with increased voting threshold.

In practice, such approaches are interested in capturing as many burnout samples as possible while maintaining a manageable number of false positives. The threshold can be modified accordingly, for example, aiming to maximize the F1 score under the condition that recall is greater than 0.9. The subreddit batching ensemble with *p* = 0.85 and the random batching ensemble with *p* = 0.5 both demonstrated performance close to such an optimum (as shown in Table 9). Both of these ensembles achieve better results than either of the baseline models.

**Table 9:** Optimal ensembles (no. test samples = 4,071).

**Model**	**Test Bal. Acc**.	**Test F1**	**Test Recall**
Subreddit Batching (*p* = 0.85)	0.93	0.43	0.93
Random Batching (*p* = 0.5)	0.93	0.42	0.93

Finally, a qualitative analysis of test samples incorrectly classified as burnout by the ensemble models revealed posts from the no-burnout dataset that contained topics similar to burnout posts, e.g., work-related, stress, depression, and anxiety. This indicates that the classifiers presented in this work are indeed identifying features related to burnout. It even appears that, in some cases, it may be the labels rather than the predictions that are incorrect, i.e., posts from scraped sub-breddits where users write about experience with burnout.

## 4. Discussion

The work presented in this article makes the following contributions:

- It demonstrates that NLP methods applied to free text are an effective means to detect indicators for burnout, measured against both a control group of general text and a group composed of text samples related to depression.
- A machine learning ensemble classifier trained on data from Reddit posts to detect burnout indicators with a promising accuracy is presented.
- A range of machine learning classifiers trained to detect burnout indicators are compared, showing in particular that the presented ensemble classifier outperforms two single classifier baselines: logistic regression classifiers trained on either a large unbalanced dataset or an undersampled balanced dataset. The best-performing model attained a balanced accuracy of 93%, F1 score of 0.43, and recall of 93% on unbalanced test data.

These findings have a large potential to be further developed with an interdisciplinary approach toward a new generation of smart tools for clinical psychology, eventually supporting a wider array of conditions and mental health diagnoses in the future.

### 4.1. Burnout Detection for a Clinical Setting

Extracting data from social media is one of the most commonly used methods in research in this area (e.g., Shen and Rudzicz, 2017; Thorstad and Wolff, 2019). The research presented in this article also relies primarily on data extracted from the social media website Reddit, particularly because it was easy to obtain a large quantity of data to train our model. Nonetheless, clinical data are a more reliable source for detecting burnout due to the certainty of labeling. Clinical data also have the advantage of more closely resembling the data such models are expected to be applied to in the future. A first attempt of working with clinical data to detect burnout has shown promising results. By presenting a dataset from real-world burnout patient data, Nath and Kurpicz-Briki (2021) managed to go beyond typical burnout detection approaches, which usually includes the use of inventories with scaling questions and worked on applying NLP to mental health. The dataset consisted of data extracted from German-language interviews with burnout patients, a control group, and experts. The authors proceeded to train an SVM classifier on the dataset and ended up achieving accuracy greater than their original baseline.

### 4.2. Burnout vs. Depression

A poorer classifier performance on Dataset 4 than on Datasets 2 or 3 was observed. This is likely due to the fact that depression- and burnout-related texts share many similar characteristics. Indeed, depression and burnout are not disjoint categories, and some degree of classification ambiguity is inevitable. This overlap is a significant object of scientific investigation, e.g., by Schonfeld and Bianchi (2016). The work in this article provides evidence of the non-trivial nature of differentiating burnout and depression. Ongoing work of the authors aims to more closely analyze the markers that indicate and differentiate depression and burnout in free text first-person accounts.

### 4.3. Methods for Dealing With Unbalanced Data

Class imbalance is a natural phenomenon in many real-world applications (e.g., fraud detection, tumor detection, software defect prediction). It is well-documented in machine learning literature that unbalanced training data impairs the classification performance of many machine learning models (e.g., Chawla et al., 2004; García et al., 2010). For example, in cases of extreme class imbalance, models can tend toward placing all samples in the majority class. For a detailed survey on the unbalanced data problem, refer to He and Garcia (2009). Class imbalance is considered to be intrinsic to the task of burnout detection from real-world (clinical) data, rather than being an artifact of the data collection methods used in this article, and, therefore, it was aimed to address the problem in this work.

Common solutions involve oversampling the minority class or undersampling the majority class to achieve class balance or using cost-sensitive methods that apply a higher penalty to the incorrect classification of samples from the minority class. A number of ensemble methods use oversampling and/or undersampling to train separate models and aggregate their predictions. Successful ensemble methods for unbalanced learning include EasyEnsemble (Liu et al., 2008), SMOTE-Boost (Chawla et al., 2003), UnderBagging (Barandela et al., 2003), and Cluster/SplitBal (Sun et al., 2015).

Sun et al. (2015) argue that most existing methods might suffer from the loss of potentially useful information and/or overfitting by altering the original data distribution. Of the ensemble methods explored in this work, only UnderBagging, ClusterBal, and SplitBal do not discard data or change the data distribution. These three methods differ mainly in how balanced data batches are constructed and how the predictions of the submodels are aggregated. The method presented in this article is most similar to that of UnderBagging, which was chosen for the ease of implementation in the given setting and the fact that (Sun et al., 2015) found that it performs well across several classifier types. The method presented in this article differs only in that different voting thresholds are considered, not all of the majority class samples are exhausted, and balanced batches based on subgroupings inherent in the presented dataset (subreddits) are constructed.

The single model experiments reflect some of the problems of class imbalance. The best classifiers trained on Dataset 1 reached lower benchmark metrics but demonstrated more consistent performance between training and test data. This is consistent with the expectation that larger training datasets generalize better. Many of the classifiers trained on the unbalanced Dataset 1 performed very poorly, essentially predicting only the majority class. On the other hand, classifiers trained on the balanced Dataset 2 attained a high benchmark performance on relatively small balanced data batches, but that performance dropped considerably (as measured by F1 scores) when applied to highly unbalanced test data. The balanced data models use undersampling and demonstrate the drawbacks of throwing out data points: much of the variance in the *no burnout* dataset is not accounted for, and the undersampling-based models incorrectly classified a relatively large number of more general *no burnout* data. As one would expect, this effect is most pronounced in the models trained using a single subreddit, where a very specialized sample of *no burnout* data were used for training.

Overall, the presented results provide evidence that both undersampling—as long as attention is paid to maintaining the variance in the majority class data—and ensemble methods are viable approaches to handling the unbalanced data problem in this context. The single logistic regression classifiers trained on undersampled, balanced data performed at a level similar to the ensembles, although the subreddit batching ensemble with *p* = 0.85 and random batching ensemble with *p* = 0.5 both outperformed the single random batching classifier in all three metrics. Undersampling does have the advantage of requiring many fewer training data with both faster training and inference, although this speed difference can be erased by running ensemble submodels in parallel. However, better performance was achieved with ensembles. The ensemble methods provide additional advantages: the voting threshold hyperparameter allows to easily fine-tune the ensemble model according to the relative importance placed on recall and F1 score; in addition, the performance of the ensemble model is more stable, i.e., immune to fluctuations according to the subsample of *no burnout* data used for training.

### 4.4. Random vs. Subreddit Batching

As similar performance with both methods for creating balanced data batches was achieved, the experiments do not indicate which, if either, of the two procedures is preferable. However, it was noted that several differences between the two methods exist. Perhaps the most important difference is that the subreddit batching ensembles required fewer training data to achieve the same performance. In addition, as Table 6 shows that the submodels in the subreddit batching ensemble achieved higher accuracy and F1 score during CV, which might result from the relative ease of distinguishing between burnout-related posts and a single specialized topic with little relation to the condition. This results in overfitting, as reflected in the gap between CV and test results recorded in Tables 6, 7. Table 7 also shows that the performance of the individual submodels in the subreddit batching ensemble varied much more than for random batching; a comparison with Table 8 also shows that the relative gain achieved by using ensemble methods over single classifiers was much greater in the case of subreddit batching. This is consistent with expectations and findings in the literature, which suggest that ensembles are an effective method for combining weak learners with considerable variance in their predictions into a strong learner (Schapire, 1990). It is also possible that the subreddits that were excluded from the ensemble due to an insufficient number of posts are over-represented among the misclassified samples and that performance could be improved by including more subreddits. Experiments in this direction are suggested for future work.

### 4.5. Recall as an Evaluation Metric

The use of recall as an evaluation metric was chosen because it is assumed that recall is of great importance in real-world applications. In the case of burnout detection, it is better to capture most or all of the true positives at the cost of a manageable number of false positives than to miss positive cases. In practice, marking individuals who are potentially experiencing burnout should help mental health professionals decide which cases should be subjected to further analysis. For this reason, even though the Baseline 1: Unbalanced LR model attained an F1 score better than or on par with the other models (Table 8), the significantly lower recall score makes this model unequivocally the least desirable. A tool that misses half of the patients demonstrating potential burnout is not useful.

### 4.6. Limitations

In this work, data procured from Reddit posts were used largely because of the ease in obtaining large quantities of data for use in model training. It is expected in the future to apply these methods to the verbal responses of patients in clinical interventions in order to train models to their destined target application. Therefore, the data origin is a limitation of this study. Obtaining a sufficient quantity (and in different local languages) of data for machine learning-based methods poses a significant challenge and will be addressed in future work by other data collection methods, involving also clinical institutions. The authors intend to collaborate with researchers and practitioners in psychology for data collection and to aid in developing a beneficial, easy-to-use clinical tool as well as expanding their work toward other areas of mental health. Another limitation of this work is the diversity in the available data. Being completely anonymous data from online forums, no information about gender, origin, socio-economic background, or similar is available. Therefore, the classifiers presented in this work may not work with the same efficiency for different groups of society. In future work, and before implementing such methods into a product, further validations and potentially additional training data will be required.

### 4.7. Future Work

In future work, the authors would like to experiment with more sophisticated ensemble methods, such as those outlined in Sun et al. (2015), where the general superiority of ensembles over other methods for addressing the class imbalance in several experiments was demonstrated. Since undersampling also showed promising results, more sophisticated methods for undersampling should be explored, such as clustering-based methods (Lin et al., 2017). However, the low variance observed among the random batching submodels may delineate the limits of undersampling-based methods. Furthermore, the use of classifier types beyond logistic regression could be explored, perhaps by incorporating neural network-based models and using other methods for creating balanced data batches for submodel training. Mixing different types of classifiers within an ensemble could be a means to capture *burnout* samples that are otherwise overlooked by logistic regression. It should also be considered to experiment with other vectorization methods in the future, particularly the use of word embeddings learned from deep learning-based language models, such as Word2Vec (Mikolov et al., 2017), GLoVe (Pennington et al., 2014), BERT (Devlin et al., 2019), and fastText (Joulin et al., 2016).

## Data Availability Statement

The datasets presented in this article are not readily available because the collected online data can be subject to change (e.g., deletions) over time. A similar dataset can be created following the instructions in the article.

## Author Contributions

MK-B is the principal investigator of the project and brought the original idea. SN was the main contributor for data collection and the first experiments, in particular, the single classifier models. AP and GM contributed by extending the single classifier models and by developing the ensemble models in collaboration with MK-B. All authors contributed to idea development, experimental setup, and paper writing/editing. All authors contributed to the article and approved the submitted version.

## Funding

The authors gratefully acknowledge the funding for this work by the Swiss National Science Foundation (SNSF) through an SNSF Spark grant.

## Conflict of Interest

The authors declare that the research was conducted in the absence of any commercial or financial relationships that could be construed as a potential conflict of interest.

## Publisher's Note

All claims expressed in this article are solely those of the authors and do not necessarily represent those of their affiliated organizations, or those of the publisher, the editors and the reviewers. Any product that may be evaluated in this article, or claim that may be made by its manufacturer, is not guaranteed or endorsed by the publisher.

## Footnotes

^1^ https://icd.who.int/browse11/l-m/en#/http://id.who.int/icd/entity/129180281

^2^ https://spacy.io/

^3^For the unbalanced dataset, a 70–30% split was taken on each class separately in order to ensure that the training and test sets had roughly the same class distribution.

^4^In our experiments, 10 ≤ *n* ≤ 20.

^5^Here, *p* is a threshold that may vary. Values of 0.4 ≤ *p* ≤ 0.99 were used.

^6^Here, we do not need to take the balanced accuracy because the submodels are trained on balanced datasets.

^7^The ensemble experiments revealed that logistic regression classifiers trained on Datasets 2 and 3 do not perform as well on a highly unbalanced data sampled from Dataset 1. This will be further discussed in Section 3.2.
